# Polymorphisms in Alcohol Metabolism Genes *ADH1B* and *ALDH2*, Alcohol Consumption and Colorectal Cancer

**DOI:** 10.1371/journal.pone.0080158

**Published:** 2013-11-25

**Authors:** Marta Crous-Bou, Gad Rennert, Daniel Cuadras, Ramon Salazar, David Cordero, Hedy Saltz Rennert, Flavio Lejbkowicz, Levy Kopelovich, Steven Monroe Lipkin, Stephen Bernard Gruber, Victor Moreno

**Affiliations:** 1 Cancer Prevention and Control Program, Catalan Institute of Oncology, Barcelona, Spain; 2 Colorectal Cancer Group, Bellvitge Biomedical Research Institute and Consorcio de Investigación Biomédica de Epidemiología y Salud Pública (CIBERESP), Barcelona, Spain; 3 Clalit Health Services, National Cancer Control Center, Department of Community Medicine and Epidemiology, Technion-Israel Institute of Technology, Haifa, Israel; 4 B. Rappaport Faculty, Medicine Carmel Medical Center, Technion-Israel Institute of Technology, Haifa, Israel; 5 Medical Oncology Service, Catalan Institute of Oncology, Barcelona, Spain; 6 Division of Cancer Prevention, National Cancer Institute, Rockville, Maryland, United States of America; 7 Department of Medicine, Weill Cornell Medical College, New York, New York, United States of America; 8 Department of Internal Medicine, Epidemiology and Human Genetics, University of Michigan Medical School, Ann Arbor, Michigan, United States of America; 9 University of Southern California Norris Comprehensive Cancer Center, Los Angeles, California, United States of America; 10 Department of Clinical Sciences, Faculty of Medicine, University of Barcelona, Barcelona, Spain; IPO, Inst Port Oncology, Portugal

## Abstract

**Background:**

Colorectal cancer (CRC) is a leading cause of cancer death worldwide. Epidemiological risk factors for CRC included alcohol intake, which is mainly metabolized to acetaldehyde by alcohol dehydrogenase and further oxidized to acetate by aldehyde dehydrogenase; consequently, the role of genes in the alcohol metabolism pathways is of particular interest. The aim of this study is to analyze the association between SNPs in *ADH1B* and *ALDH2* genes and CRC risk, and also the main effect of alcohol consumption on CRC risk in the study population.

**Methodology/Principal Findings:**

SNPs from *ADH1B* and *ALDH2* genes, included in alcohol metabolism pathway, were genotyped in 1694 CRC cases and 1851 matched controls from the Molecular Epidemiology of Colorectal Cancer study. Information on clinicopathological characteristics, lifestyle and dietary habits were also obtained. Logistic regression and association analysis were conducted. A positive association between alcohol consumption and CRC risk was observed in male participants from the Molecular Epidemiology of Colorectal Cancer study (MECC) study (OR = 1.47; 95%CI = 1.18-1.81). Moreover, the SNPs rs1229984 in *ADH1B* gene was found to be associated with CRC risk: under the recessive model, the OR was 1.75 for A/A genotype (95%CI = 1.21-2.52; p-value = 0.0025). A path analysis based on structural equation modeling showed a direct effect of *ADH1B* gene polymorphisms on colorectal carcinogenesis and also an indirect effect mediated through alcohol consumption.

**Conclusions/Significance:**

Genetic polymorphisms in the alcohol metabolism pathways have a potential role in colorectal carcinogenesis, probably due to the differences in the ethanol metabolism and acetaldehyde oxidation of these enzyme variants.

## Introduction

Colorectal cancer (CRC) is a leading cause of death worldwide, with over one million new cases and half a million deaths around the world every year [1], [2]. Risk factors for CRC include advanced age, medical history of benign adenomatous polyps and inflammatory bowel diseases, family history of CRC, low intake of vegetables and fruits and high intake of dietary fat (particularly animal fat) and processed meat [3], [4], [5], [6]. Chronic consumption of non-steroidal anti-inflammatory drugs, hormone replacement therapy and statins are protective [7], [8]. The role of other lifestyle factors such as tobacco smoking [9], [10] or alcohol consumption [11], [12], [13], [14], [15], [16] remains inconclusive. Alcohol consumption has been reported to be associated with modest increased risks of CRC in some studies [17], but cancer risk may differ by tumor molecular subtype and anatomical site.

Although the mechanism by which alcohol influences CRC risk also remains not well understood [18], different hypothesis have been suggested: a carcinogenic effect of chemicals other than ethanol present in alcoholic beverages such as nitrosamines, a solvent action than facilitates absorption of other carcinogens, an inhibition of methylation caused by ethanol, or a carcinogenic and genotoxic role for acetaldehyde, the major metabolite of ethanol [19], [20]. There is sufficient evidence for the carcinogenicity of ethanol in experimental animals, and it is also considered carcinogenic to humans. Especially, there is evidence on the fact that drinking of alcoholic beverages is causally related to cancers of the oral cavity, pharynx, larynx and liver (IARC Group1); However, there is controversial evidence on the effect of alcohol consumption on gastric or colorectal cancer [21]. Moreover, there is increasing evidence that acetaldehyde, a cytotoxic, mutagenic, and carcinogenic metabolite of ethanol, is responsible for tumor enhancing effects leading to aberrant cell proliferation. Acetaldehyde can also bind to DNA, leading to the formation of stable DNA adducts and the generation of reactive oxygen species (ROS) that may cause replication errors and mutations in oncogenes and tumor suppressor genes [19], [22], [23], [24], [25]. Moreover, some studies have reported epigenetic alterations by alcohol metabolites including selective acetylation, methylation, and phosphorylation of histones that regulate gene expression during disease pathogenesis [26], [27], [28].

The amount of acetaldehyde present in various tissues following ethanol ingestion may not only depend on the amount of ethanol consumed but also on specific alleles of genes coding for ethanol-metabolizing enzymes. Ethanol is metabolized to acetaldehyde mainly by alcohol dehydrogenase (ADH) enzyme, and is further oxidized to acetate by acetaldehyde dehydrogenase (*ALDH*) (Supporting file Figure S1). Single nucleotide polymorphisms (SNPs) of *ADH1B* and *ALDH2*, the genes that encodes the key enzymes in alcohol metabolism, might cause variations in the amount of production and/or oxidation of acetaldehyde between individuals [18], [19], [29]. Therefore, polymorphisms in genes involved in alcohol metabolism pathways might influence CRC susceptibility.

The aim of the present study is to assess the association between SNPs in alcohol metabolism genes, in particular in *ADH1B* and *ALDH2* genes, and CRC, and the main effect of alcohol consumption on CRC risk in the study population. The analyses have been performed within a large population-based case-control study conducted in Israel, the Molecular Epidemiology of Colorectal Cancer study (MECC) study.

## Methods

### Patients

The MECC study has already been described in detail [8], [30]. In brief, it is a population based case-control study of incident, pathologically confirmed, invasive CRC diagnosed between May 31, 1998 and March 31, 2004 and who lived in a geographically-defined area of Northern Israel.

Controls with no prior history of CRC were identified from the same source population using the Clalit Health Services (CHS) database, covering ∼70% of the older population. Controls were matched to cases by year of birth, gender, primary clinic location, and Jewish or Arab ethnicity. Participants provided written informed consent at the time of enrollment. The Institutional Review Boards at the Carmel Medical Center, the University of Michigan, and the University of Southern California approved all procedures. The study population included 2100 matched pairs. For this analysis, 1780 cases and 1864 controls with available DNA for genetic analysis will be used. The demographic characteristics of the subjects not included do not differ from those analyzed (data not shown).

### Life-style information

Participants were interviewed face-to-face by trained monitors with a comprehensive epidemiological questionnaire that assessed demographic information, personal and family history of cancer, reproductive history and medical history, medication use, health habits and a food-frequency questionnaire.

Patient’s weight was recorded by self-report, as estimated one year before diagnosis for cases and at the time of interview for controls. Body mass index (BMI) was estimated one year before the diagnosis from self-reported weight and height.

Comprehensive dietary habits were obtained with the use of a validated food-frequency questionnaire modified for the Israeli diet. Alcohol frequency was registered as the alcohol consumption for the past five years before CRC diagnosis; beer, wine and other hard liquor were recorded separately (the question was exactly “have you ever consumed beer and/or wine and/or other hard liquor at least once a weak for at least one year?”). Alcohol consumption was converted to a categorical variable that includes three levels of consumption: non-drinkers, occasional drinkers (participants who only drink wine for the blessing) and drinkers. The prevalence of alcohol consumption in the study population is low, like in other Jewish population [31], [32]. Total fat and vegetable intakes were estimated from the questionnaire using local food composition tables. Similar estimates provided total energy consumption.

Physical activity was recorded for the longest occupation and also recreational physical exercise. These were combined into an ordinal index ranging from sedentary to intense physical activity.

### SNP selection and genotyping

SNPs in two candidate genes related to key alcohol metabolism pathways, specifically rs1229884 in *ADH1B* gene and rs886205 in *ALDH2*, were selected for analysis within a larger exploratory project including other hypothesis. The SNPs were selected as maximally informative htSNPs when the regions were analyzed with HaploTagger. SNPs were genotyped using Illumina BeadStation and BeadExpress platforms. Data were analyzed using unsupervised Illumina BeadStation SNP calling automated routines, and the distribution of genotypes was compared to those expected from Hardy-Weinberg equilibrium. Genotypes were also confirmed with a second platform in at least 1% of samples.

### Statistical Methods

Unconditional logistic regression was used to assess the association between genotypes and CRC risk. All models included age and gender to account for the matching design and avoid excluding incomplete pairs due to DNA unavailability or missing values in other variables. The total number of cases and controls with both, lifestyle information and genetic data available for this analysis were 1694 and 1851, respectively. Baseline characteristics did not differ to the complete dataset. Analyses regarding alcohol have been restricted to men due to the low prevalence of alcohol consumption among the female population.

To estimate the magnitude of the associations, multivariate-adjusted odds ratios (OR) and their corresponding 95% confidence intervals (CI) were calculated for each genotype compared to those homozygous for the common allele. Also a log-additive model was fitted (trend test). Genetic analysis were performed with the SNPstats web software [33] and SNPassoc library from R package [34]. A panel of 300 anonymous SNPs useful for population stratification analysis was analyzed in these subjects in relation to other larger genotyping project. No relevant population structure was identified that could not be explained by reported ethnicity. Although the potential confounding of this variable was rejected after a sensitivity analysis (data not shown) it was also included in the models as a potential confounder.

A global 5% significance level was desired for the analyses of SNPs. All reported p-values are two-tailed, and adjusted for age, sex, ethnicity, energy intake and physical activity. Other variables were assessed as potential confounders (i.e., BMI, vegetable consumption, fat intake, red meat consumption) but were not included in the modes since they did not significantly alter the estimates (results not shown).

To simultaneously study the associations between SNPs, potential confounding variables and CRC we also fitted structural equation models (SEM) to assess the direct effect of genetic polymorphisms on CRC (unexplained by candidate variables), and also their indirect effect, mediated through lifestyle related factors, by including all variables in the same model to assess overall associations. These analyses were performed using the structural equation modeling library from R package. SEM was used to test the conceptual model since, in contrast to traditional analytical procedures as linear regression analysis, SEM allows distinguishing between direct and indirect effects and provides information on the degree of fit for the entire model. In SEM the covariance structure that follows from the proposed model is fitted to the observed covariance. The maximum likelihood estimate method yields estimates of the regression coefficients in the model, standard errors and an overall goodness-of-fit test [35].

## Results

The study population included 3545 participants, 1694 cases and 1851 controls, with available data regarding epidemiological interviews (including alcohol consumption) and genetic analysis. A description of the subjects’ characteristics is in Table 1. Cases had larger reported weight and BMI one year before the diagnosis than controls. Cases also reported more family history of CRC and lower physical activity and vegetable intake than controls, and were more often Ashkenazi than Sephardi.

**Table 1:** Baseline characteristics of cases and controls from the study population.

Clinical/Baseline characteristics	All participants			Men only		
	Controls	Cases	p-value	Controls	Cases	p-value
N (%)	1864 (51)	1780 (49)		931 (51)	894 (49)	
**Age** - mean (SD)	70.6 (11.7)	70.2 (11.7)	0.34	71.3 (11.3)	70.9 (11.3)	0.40
**Sex**			0.87			
Male - N (%)	931 (50)	894 (50)				
Female - N (%)	933 (50)	886 (50)				
**BMI** - mean (SD)	26.9 (4.6)	27.3 (4.6)	0.027	26.4 (3.7)	27.1 (4.1)	0.00058
**History of colorectal cancer** in first-degree relative			0.0018			0.12
No - N (%)	1722 (93)	1571 (90)		866 (93)	806 (91)	
Yes - N (%)	132 (7)	175 (10)		61 (7)	75 (9)	
**Physical activity**			<0.0001			0.0011
Sedentary	1105 (62)	1231 (69)		516 (56)	571 (64)	
Intermediate	447 (25)	311 (18)		226 (24)	173 (19)	
Active	241 (13)	238 (13)		189 (20)	150 (17)	
**Ethnicity**			<0.0001			0.0009
Ashkenazi	1176 (63)	1249 (70)		595 (64)	633 (71)	
Sephardi	445 (24)	297 (17)		219 (23)	149 (17)	
Other	241 (13)	234 (13)		117 (13)	112 (12)	
**Religion**			0.33			0.94
Jew	1651 (89)	1576 (88.54)		828 (88.94)	796 (89.04)	
Christian	91 (5)	102 (5.73)		49 (5.26)	49 (5.48)	
Moslem	122 (6)	102 (5.73)		54 (5.8)	49 (5.48)	
**Vegetable intake (g/day) - mean (SD)**	7.9 (3.7)	7.5 (4.5)	0.0061	8.1 (3.7)	7.6 (4.1)	0.0026
**Regular aspirin intake**						
No - N (%)	1170 (63)	1192 (70)	<0.0001	524 (56)	558 (64)	0.0001
Yes - N (%)	692 (37)	531 (30)		406 (44)	317 (36)	
**Regular statin intake**						
No - N (%)	1623 (88)	1630 (94)	<0.0001	795 (86)	819 (93)	<0.0001
Yes - N (%)	228 (12)	101 (9)		128 (14)	59 (7)	
**Energy intake** (Kcal/day) - mean (SD)	1856 (1114)	1889 (1587)	0.48	1996 ( 1249)	2058 (1878)	0.41
**Total fat intake** (g/day) - mean (SD)	66.1 (44.9)	69.3 (67.5)	0.0968	71.3 (50.4)	76.6 (81.8)	0.1016
**Smoking**						
Never smoker- N (%)	1035 (56)	993 (57)	0.38	327 (35)	323 (37)	0.53
Ever smoker - N (%)	826 (44)	747 (43)		603 (65)	560 (63)	
**Alcohol consumption**, past five years						
No or occasionally- N (%)	1587 (85)	1405 (81)	0.0002	693 (75)	599 (68)	0.0013
Yes - N (%)	272 (15)	336 (19)		235 (25)	284 (32)	

Though energy intake was similar for cases and controls, total fat intake was larger for cases than controls. Long term regular use of aspirin and statins were also inversely associated with CRC risk. Smoking habits were similar in cases and controls, while higher proportion of cases reported alcohol consumption for the five years before diagnosis than controls (p<0.001).

A description of subjects’ characteristics by categories of alcohol consumption is in Table 2. Only 17% of participants reported alcohol consumption (other than wine for the blessing) for the past five years. No differences were observed regarding reported BMI, ethnicity, long term use of aspirin and statins and family history of CRC. Alcohol drinkers were mainly men, Christian religion, and had higher vegetables, fat and energy intakes. A large difference of alcohol consumption prevalence was observed between men and women. Since the prevalence of alcohol drinking in women was very low (<5%), women were excluded from the association analysis. Baseline characteristics of men’s population included in the study is shown in Table 1, and also by alcohol consumption in Table 2.

**Table 2:** Distribution of participant characteristics by categories of alcohol consumption.

Clinical/Baseline characteristics	Alcohol consumption (all participants)			Alcohol consumption (men only)		
	No or Occasional	Yes	p-value	No or Occasional	Yes	p-value
N (%)	2992 (83)	608 (17)		1292 (71)	519 (29)	
**Age** - mean (SD)	70.5 (11.7)	69.4 (11.3)	0.028	71.59 (11.37)	69.87 (11.10)	0.0032
**Sex**			<0.0001			
Male - N (%)	1292 (43)	519 (85)				
Female - N (%)	1700 (57)	89 (15)				
**BMI** - mean (SD)	27.1 (4.8)	26.7 (3.7)	0.016	26.7 (4.1)	26.7 (3.5)	0.82
**History of colorectal cancer** in first-degree relative			0.96			0.45
No - N (%)	2707 (91)	552 (91)		1186 (93)	473 (92)	
Yes - N (%)	253 (9)	52 (9)		93 (7)	43 (8)	
**Physical activity**			<0.0001			<0.0001
Sedentary	1990 (66)	307 (51)		815 (63)	261 (50)	
Intermediate	593 (20)	141 (23)		274 (21)	124 (24)	
Active	409 (14)	160 (26)		203 (16)	134 (26)	
**Ethnicity**			0.25			0.14
Ashkenazi	1977 (66)	413 (68)		868 (67)	351 (68)	
Sephardi	627 (21)	110 (18)		272 (21)	93 (18)	
Other	388 (13)	85 (14)		152 (12)	75 (14)	
**Religion**			<0.0001			<0.0001
Jew	2647 (88)	538 (89)		1155 (89)	457 (88)	
Christian	135 (5)	57 (9)		48 (4)	49 (9)	
Moslem	210 (7)	13 (2)		89 (7)	13 (3)	
**Vegetable intake** (g/day) - mean (SD)	7.5 (4.1)	8.5 (4.1)	<0.0001	7.6 (3.9)	8.4 (4.0)	0.0003
**Regular aspirin intake**						
No - N (%)	1977 (66.2)	378 (62.3)	0.17	763 (59.3)	315 (60.8)	0.12
Yes - N (%)	994 (33.3)	225 (37.1)		522 (40.6)	199 (38.4)	
**Regular statin intake**						
No - N (%)	2700 (91)	540 (90)	0.25	1147 (90)	459 (89)	0.66
Yes - N (%)	265 (9)	63 (10)		130 (10)	56 (11)	
**Energy intake** (Kcal/day) - mean (SD)	1811 (931)	2178 (1022)	<0.0001	1957 (1042)	2200 (1031)	0.0059
**Total fat intake** (g/day) - mean (SD)	65.1 (53.5)	80.9 (70.1)	<0.0001	70.8 (65.1)	81.8 (72.7)	0.0030
**Smoking**						
Never smoker- N (%)	1853 (62)	170 (28)	<0.0001	528 (41)	120 (23)	<0.0001
Ever smoker - N (%)	1132 (38)	438 (72)		762 (59)	399 (77)	
**Participant status**						
Control- N (%)	1587 (53)	272 (45)	0.0002	693 (54)	235 (45)	0.0013
Case - N (%)	1405 (47)	336 (55)		599 (46)	284 (55)	

### Alcohol consumption and CRC risk

Although the prevalence of alcohol consumption in the study population was low, and only 29% of men reported alcohol consumption (other than wine for the blessing) for five years before CRC diagnostic, the prevalence of alcohol consumption was higher in male cases than in male controls (p<0.01) (Table 1). The odds ratio (OR) adjusted for age, ethnicity, religion, energy intake and physical activity was 1.47 (95%CI = 1.18-1.81; p = 0.0004).

### Alcohol metabolism SNPs and CRC risk

SNPs in 2 candidate genes related to key alcohol metabolism pathways, specifically rs1229884 in *ADH1B* gene and rs886205 in *ALDH2*, were genotyped in CRC cases and controls and selected for the present analysis. A detailed description of the allele and genotype frequencies for the studied SNPs is shown in Supporting Information file Table S1. Details of the association between *ADH1B* and *ALDH2* polymorphisms and CRC risk are shown in Table 3. Except for those indicated, ORs were adjusted for age, ethnicity, religion energy intake and physical activity, and the association analysis was restricted to men. The model of inheritance presented in tables was selected on the basis of the lowest value of Akaike's information criterion.

**Table 3:** Association between polymorphisms in alcohol metabolism genes and colorectal cancer risk (men only).

SNP	Controls	Cases	OR^1^	95% CI	p-value
	N (%)	N (%)			
**ADH1B rs1229984**	927 (51.9)	860 (48.1)			0.0096
G/G	513 (55.3)	457 (53.1)	1.00		
G/A	360 (38.8)	324 (37.7)	1.04	(0.85–1.26)	
A/A	54 (5.8)	79 (9.2)	1.77	(1.22–2.58)	
Recessive (A/A)	54 (5.8)	79 (9.2)	1.75	(1.21–2.52)	0.0025
**ALDH2 rs886205**	916 (51.8)	854 (48.2)			0.61
T/T	658 (71.8)	637 (74.6)	1.00		
T/C	228 (24.9)	189 (22.1)	0.91	(0.73–1.15)	
C/C	30 (3.3)	28 (3.3)	1.16	(0.67–1.99)	
Dominant (T/C-C/C)	258 (28.2)	217 (25.4)	0.94	(0.76–1.17)	0.57

1 OR adjusted for age, ethnicity, religion, energy intake and physical activity.

An association was observed between rs1229984 polymorphism in *ADH1B* and CRC risk. The A allele of this SNP was associated with an increased risk of CRC. Under the recessive model, the multi-adjusted OR was 1.75 for A/A genotype (95%CI = 1.21-2.52, p-value = 0.0025).

No association between rs886205 polymorphism in *ALDH2* and CRC risk was observed. Under the dominant model, the OR adjusted for potential confounders was 0.94 for T/C and C/C genotypes (95%CI = 0.76-1.17, p-value = 0.57). No significant interaction between *ADH1B* and *ALDH2* polymorphisms was observed (p = 0.89; data not shown).

### Alcohol metabolism SNPs and alcohol consumption

The association between *ADH1B* and *ALDH2* polymorphisms and alcohol consumption in men controls is shown in Table 4. The recessive model for polymorphism rs1229984 in *ADH1B* showed a non-significant association with alcohol consumption (OR = 1.79, 95%CI = 0.97-3.28, p =  0.068). However, polymorphism rs886205 in *ALDH2* was not associated to alcohol consumption (OR = 1.06, 95%CI = 0.75-1.50, p = 0.75). No significant interactions were observed between *ADH1B* polymorphisms and alcohol consumption or between *ALDH2* polymorphisms and alcohol consumption (p = 0.73 and p = 0.81, respectively; data not shown).

**Table 4:** Association between polymorphisms in alcohol metabolism genes and alcohol consumption (men only).

SNP	Alcohol consumption		OR^1^	95% CI	p-value
	No or Occasional	Yes			
	N (%)	N (%)			
**ADH1B rs1229984**	690 (74.7)	234 (25.3)			0.10
G/G	378 (54.8)	133 (56.8)	1.00		
G/A	276 (40.0)	83 (35.5)	0.83	(0.60–1.15)	
A/A	36 (5.2)	18 (7.7)	1.66	(0.89–3.08)	
Recessive (A/A)	36 (5.2)	18 (7.7)	1.79	(0.97–3.28)	0.068
**ALDH2 rs886205**	682 (74.7)	231 (25.3)			0.69
T/T	493 (72.3)	164 (71.0)	1.00		
T/C	166 (24.3)	62 (26.8)	1.10	(0.77–1.57)	
C/C	23 (3.4)	5 (2.2)	0.72	(0.25–2.02)	
Dominant (T/C-C/C)	189 (27.7)	67 (29.0)	1.06	(0.75–1.50)	0.75

1 OR adjusted for age, ethnicity, religion, energy intake and physical activity.

### Path analysis of SNPs, alcohol consumption, physical activity, energy intake, ethnicity and CRC

The main aim of this analysis was to perform, beyond the association study, a combined analysis of all players in alcohol metabolism. Path analysis using structural equation models (SEM) provide an interesting tool for this, since a simultaneous estimate of associations can be done for an a priori proposed model. We built the model from the observed associations in the multiple univariate analyses previously described and tried to fit SEM to identify strong association resistant to adjustment for indirect effects.

**Figure 1:**
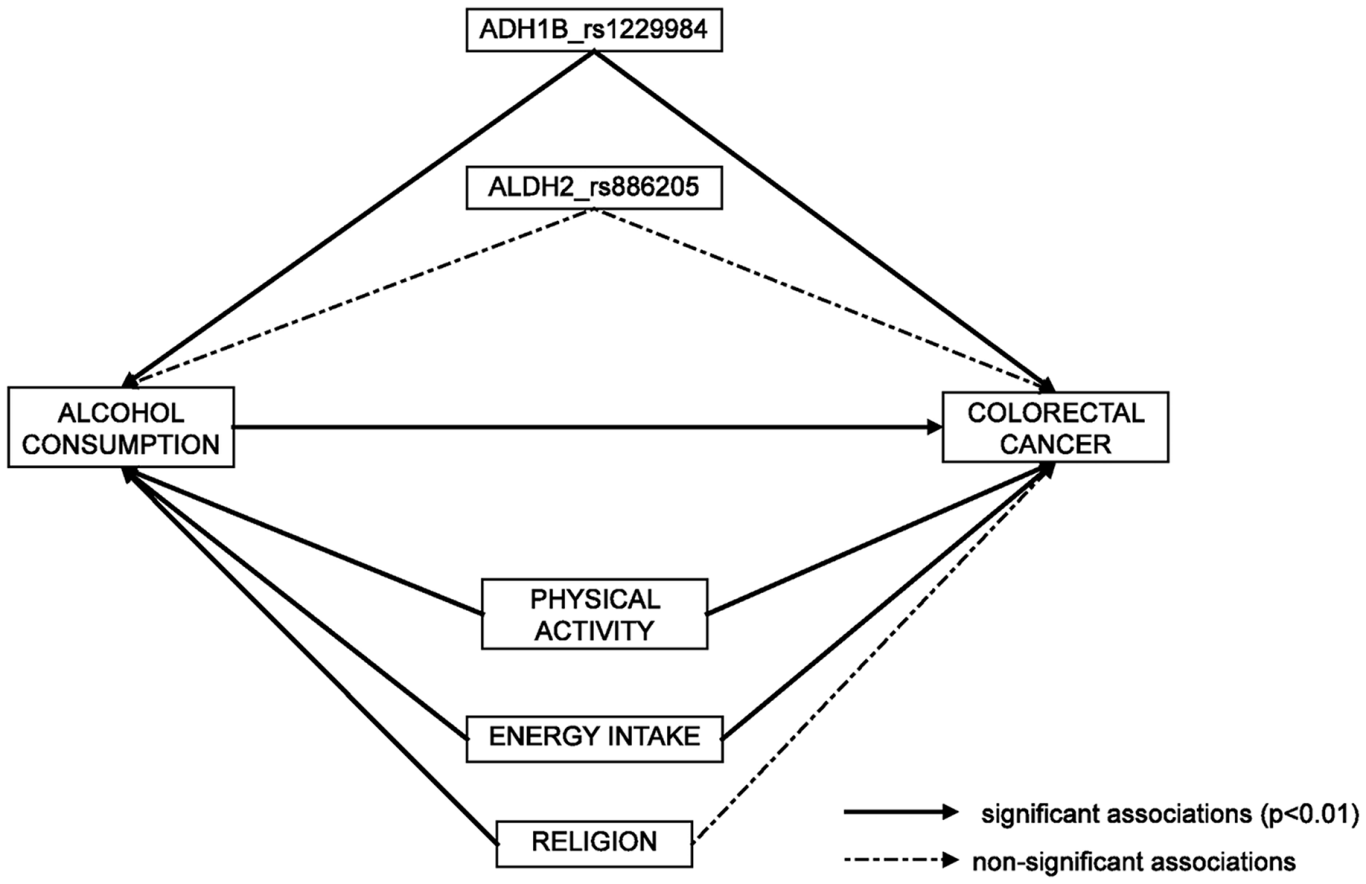
Polymorphisms in alcohol metabolism genes ADH1B and ALDH2, alcohol consumption and colorectal cancer: path analysis using Structural Equation Modeling (SEM). Continuous arrows indicate significant associations (p-value < 0.01) and discontinuous arrows indicate non-significant associations.

Figure 1 shows the proposed model reduced to relevant associations. The continuous arrows show the associations that remain significant in the SEM. Discontinuous arrows show associations that were no longer significant and could be considered as indirect effects. This analysis confirms the direct effect of *ADH1B* on CRC and also an indirect effect of such polymorphisms through alcohol consumption. Behavioral variables, like energy intake and physical exercise, also remain related among each other and associated to CRC.

## Discussion

We have examined the association between alcohol consumption, polymorphisms in alcohol metabolism genes and the risk of CRC. An increased risk of CRC was observed in alcohol drinkers. Polymorphism rs1229984 in *ADH1B* has been found to be directly associated with CRC risk and it also shows an indirect effect, mediated through alcohol consumption, even when energy intake, physical activity and religion are included in the model as potential confounders. No association between *ALDH2* polymorphism rs886205 and CRC risk was observed, neither by the indirect effect of such polymorphisms on alcohol consumption.

In humans, the major enzymes involved in the alcohol metabolizing pathways are alcohol dehydrogenase-1B (*ADH1B*) and aldehyde dehydrogenase-2 (*ALDH2*). *ADH1B*, that is expressed in the liver and present in gastrointestinal tract, metabolize ethanol to acetaldehyde that is further oxidized to acetate by *ALDH2* [19]. The activity of these enzymes varies between individuals, so genetic polymorphisms in *ADH1B* and *ALDH2* genes could be responsible for differences in the expression and activity of the enzymes as well as the subsequent metabolites generated by the enzymes. Polymorphisms in genes responsible for these pathways can affect the amount of acetaldehyde and reactive oxygen species generated during the metabolic process, altering the effects of alcohol, and potentially leading to carcinogenesis [36], [37].

It has been reported that after alcohol absorption, the concentration of alcohol in the colon is higher than in the blood. In animal experiments, acetaldehyde concentration in the mucosa of large intestine could exceed the concentration that is considered to be mutagenic. Moreover, when aldehyde dehydrogenase activity was inhibited, tumorigenesis was observed in the colon of rats. In addition, bacteria in the large intestine can also metabolize alcohol into acetaldehyde, but do not metabolize acetaldehyde to acetic acid for detoxification, so this cytotoxic metabolite could accumulate and reach high concentrations in the colon [19], [38], [39], [40].

Previous studies have reported associations between polymorphisms of alcohol metabolizing enzymes and CRC risk, mainly in oriental population, but results are inconclusive since associations reported are not always replicated by other studies [36], [37], [41], [42], [43], [44], [45]. The most frequently reported loci are *ADH1B* Arg47His (rs1229984), because the activity of *ADH1B* decreased by 40-fold in *ADH1B* His/His individuals, and *ALDH2* Glu487Lys (rs671), which affects the Km of this enzyme for alcohol with loss of the enzyme activity in individuals with the *ALDH2* Lys/Lys phenotype. However, most of these studies were focused on the functional loci reported in other diseases; only the study of Yang et al, 2009 [36] reported a tagSNP-based association study to investigate novel loci that have not been reported before, including the ones analyzed in our study, rs1229984 SNP of *ADH1B* gene and rs886205 of *ALDH2* gene. In this hospital-based case-control study of 440 CRC patients and 800 cancer-free controls in Southwestern Chinese population, none of these 2 SNPs were significantly associated with CRC risk. Recently, Ferrari et al. [44] evaluated the impact of rs1229984 polymorphisms and CRC risk in a nested case-control study (1269 cases and 2107 matched controls) within the European Prospective Investigation into Cancer and Nutrition study (EPIC) and they founded that the SNP was associated with a reduction in alcohol consumption but not with CRC risk overall in Western-European Populations.

Our results showed direct effect of *ADH1B* gene polymorphisms on colorectal carcinogenesis and also a weak indirect effect of such polymorphisms mediated through alcohol consumption, even including energy intake and physical activity in the models as potential confounders. Since polymorphisms in *ADH1B* gene contributed in the increased risk of CRC, the role of this enzyme in colorectal carcinogenesis could be due to its efficiency in ethanol metabolization and acetaldehyde detoxification and to the amount of acetaldehyde that is consequently accumulated leading to colorectal carcinogenesis.

This study is one of the largest conducted so far in which polymorphisms in alcohol metabolism genes have been studied in the global context of energy intake and physical activity in relation to CRC. The population-based case-control design, though retrospective, has revealed useful to identify a series of associations that allow a global picture of the key players. Some of the reported associations had been previously reported in studies of specific factors, which reinforces the strength of the proposed model. The path analysis using structural equations models, though not novel, is a good tool to provide a comprehensive analysis of all the players in the putative causal model. This analysis has identified direct and mediated effects of alcohol-related factors and CRC risk. The study also has some limitations. Most important ones are that alcohol intake was assessed only by self-report questionnaire, which may be liable to reporting biases. The population under study is characterized by a low prevalence of alcohol consumption, mostly related to religious traditions among Jewish and Arabs. This has impacted in a reduced sample size in the alcohol-exposed categories. Also, the polymorphisms studied have been reduced to one SNP per gene, which may not be enough to capture all the genetic variability.

## Conclusion

Our results suggest a role of polymorphism rs1229984 in *ADH1B* in colorectal carcinogenesis but have failed to show such association for polymorphism rs886205 in *ALDH2*. Although our findings require validation in other independent population, further characterization of *ADH1B* and *ALDH2* polymorphisms may provide new insights into their contribution to incidence and progression of CRC.

## Supporting Information

Figure S1 **Role of** ***ADH1B*** **and** ***ALDH2*** **in ethanol metabolism.**
(TIF)

Table S1 **Allele and genotype frequencies for the studied SNPs.**
(DOCX)
