# Supplementary figures and images for: Polymorphisms in Alcohol Metabolism Genes ADH1B and ALDH2, Alcohol Consumption and Colorectal Cancer
Source: PLoS One. 2013 Nov 25;8(11):e80158. doi: 10.1371/journal.pone.0080158 (PMC3839967; doi:10.1371/journal.pone.0080158)

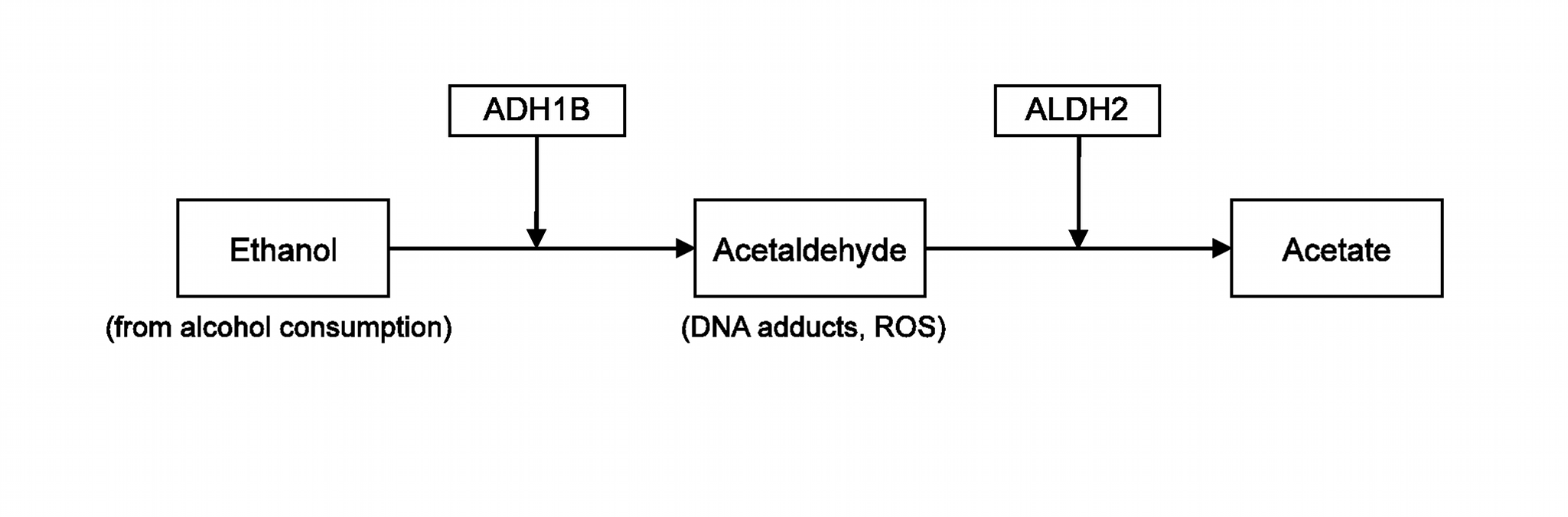

Supplement: Figure S1 — Role of ADH1B and ALDH2 in ethanol metabolism. (TIF) [file pone.0080158.s001.tif]
