# Supplementary material for: Polymorphisms in Alcohol Metabolism Genes ADH1B and ALDH2, Alcohol Consumption and Colorectal Cancer
Source: PLoS One. 2013 Nov 25;8(11):e80158. doi: 10.1371/journal.pone.0080158 (PMC3839967; doi:10.1371/journal.pone.0080158)
Supplement: Table S1 — Allele and genotype frequencies for the studied SNPs. (DOCX) [file pone.0080158.s002.docx]

| **Supplementary table 1. Description of the allele and genotype frequencies for the studied SNPs and p-value for the Hardy-Weinberg equilibrium in the control group of the study population.** | | | | |
| --- | --- | --- | --- | --- |
| **SNP** | **Allele/Genotype frequencies** | **Allele/Genotype frequencies** | **Allele/Genotype frequencies** | **Hardy-Weinberg equilibrium**  **(controls)** |
|  | **All subjects** | **Controls** | **Cases** | **p-value** |
|  | **N (%)** | **N (%)** | **N (%)** |  |
| **ADH1B rs1229984** |  |  |  | 1.00 |
| G | 5299 (72.4) | 2743 (73.86) | 2556 (72) |  |
| A | 1965 (27.05) | 971 (26.14) | 994 (28) |  |
| G/G | 1904 (53.41) | 1013 (54.55) | 927 (52.22) |  |
| G/A | 1419 (39.07) | 717 (38.61) | 702 (39.55) |  |
| A/A | 273 (7.52) | 127 (6.84) | 146 (8.22) |  |
| **ALDH2 rs886205** |  |  |  | 0.23 |
| T | 6038 (84.12) | 3050 (83.47) | 2988 (84.79) |  |
| C | 1140 (15.88) | 604 (16.52) | 536 (15.21) |  |
| T/T | 2565 (71.47) | 1280 (70.06) | 1285 (72.93) |  |
| T/C | 908 (25.30) | 490 (26.82) | 418 (23.72) |  |
| C/C | 116 (3.23) | 57 (3.12) | 59 (3.35) |  |
